# A Case of Tetanus

**Published:** 1851-02

**Authors:** W. H. Cobb

**Affiliations:** Louisville, Ky.


Art. III.—*A Case of Tetanus.* Reported by W. H. Cobb, M. D., of Louisville, Ky.

The subjoined case occurred in the surgical ward of the Louisville Marine Hospital, while under the charge of Prof. Paul F. Eve, of the University, to whose courtesy I am indebted for the frequent opportunities I enjoyed, of watching the progress of the case:

The patient, John Haynes, is an Irishman by birth, 30 years of age, of a strong, robust frame and healthy constitution. About the first of December, while engaged in blasting rock on the railroad between New Albany and Salem, Ia., the powder exploded as he was in the act of bending over the charge. Both eyes were irretrievably destroyed; his face horribly mangled and bruised, and fragments of the rock deeply imbedded in its structure. In addition, a severe lacerated wound was inflicted upon the palmar surface of the little and ring fingers, and the ulnar border of the palm of the left hand; the tendons, nerves and blood-vessels supplying these structures were laid bare, and in some places the bone was completely denuded. A comrade, Higgins by name, assisting him in his operations, also sustained a loss of both eyes, fracture of the inferior maxilla and other injuries of an apparently more serious character than the patient whose case I am about to lay before you. They entered the Hospital Dec. 10th.

The treatment adopted, both prior and subsequent to their entrance, was mildly antiphlogistic; removal of foreign substances, exhibition of mild laxatives whenever the state of the bowels seemed to indicate them, the use of poultices and warm water dressings to the wound, restriction as to diet, &c. Within the last few days, the wound on the hand of Haynes has been dressed with a solution of chloride of soda—one part of "Labarraque's Disinfecting Solution" to 13 of water.

Dec. 18th. Patient doing well, appetite much improved, rests well at night. Complains of a slight increase of the pain in the eyes. Bowels costive. Ordered:

℞. Ol. Ricini ℥j.

19th. M. Dr. Eve's attention was, for the first time, directed by the patient to the existence of convulsive twitchings, involving the muscles of the entire left forearm. Upon questioning the nurse of the ward, he stated that yesterday, while dressing the wound of the hand, he perceived a small fragment of bone protruding from its surface; simultaneously with its removal, the patient experienced spasm of the fore arm of the affected side. The spasms recurred at long intervals during the day, whenever any of the exciting causes were in action; at other times the muscles were perfectly quiet. The patient also complained of soreness and pain about the muscles of the jaw, but upon instituting an examination, the irritation was found to be owing to a carious tooth.

Actual condition at time of visit:

The patient is suffering from tetanic spasms, recurring at intervals of about 1 minute, as yet mild in their character, and affecting only the left forearm. No pain or uneasiness about the jaws, muscles of the neck, or diaphragm; opens his mouth with ease, and converses without difficulty; mind clear, pulse, skin and tongue natural. Bowels freely evacuated by the Castor oil. The patient expresses himself as feeling "well, barring these jerks."

The tetanic spasms evidently arise from the lacerated palmar wound, but no foreign matters or other source of irritation can be detected. The wound itself is in a state of unhealthy suppuration with a slight tendency to a sloughing condition of its margins; the pus thin and sanious, the granulations studding the center, pale, flabby and exuberant; integuments of the arm and forearm perfectly healthy.

Ordered—100 drops of Tinct. Opii—to be repeated in the dose of 50 drops every half hour, till the spasms subside or narcotism be induced. Locally—

℞. Pulv. Opii ʒj.

Aquæ bullientis ℥iv.

M. ft. infusion, to be added to a flaxseed poultice, and applied warm to the wound. A blister, eight inches by four, to the nape of the neck.

4 o'clock, P.M. Visited the patient, and found him perfectly easy, with an entire intermission of the symptoms; had slept soundly during the afternoon, but was awake at the time of visit. Opiate treatment directed to be continued.

20th.—2 o'clock. The intermission of the spasms continued till 3 o'clock this morning—an interval of about fifteen hours since relief was first afforded the patient. He dosed at intervals during the night, but his slumber was disturbed by horrible dreams of suffocation and drowning. At 3 o'clock, A.M., as before mentioned, the symptoms recurred, at first so mild and transient in their character that the patient did not deem them worth mentioning, though expressly requested to do so. It was not until 5 o'clock that they became so painful as to induce the patient to call upon the house surgeon, Dr. Selby.

Finding the opiate treatment ineffectual, the patient was directed to take the following, internally:

℞. Fol. Tabaci ʒj,

Aq. bull., ℥iv.

M. ft. infusion.

℥j every half hour, to complete relaxation of the system.

Present condition. Skin and pulse natural; mind perfectly clear. Paroxysms of contraction affecting the whole body, of an intensity and violence far exceeding those of any previous period. No well marked emprosthotonos or opisthotonos. Muscles of the upper extremities more implicated than those of the rest of the body; patient opens his mouth and swallows without much difficulty.

The whole of the infusum tabaci, as prescribed, was taken without any sensible effect, either upon the disease, or upon the state of the system generally.

The patient was then directed to inhale chloroform till complete anæsthesia be produced, and to repeat it whenever the spasms recurred. Also to take internally,

℞. Tinct. assafetidæ, } 100 gtt.

Tinct. opii, }

and repeat the same in the dose of fifty drops of each, every half hour.

21st.—The tetanic symptoms began to remit about 8 o'clock in the afternoon of yesterday, and at 9 o'clock ceased entirely. He dozed in a restless state during the night, but enjoyed no sound and refreshing sleep. The medicine was administered to him regularly during the night, except when dozing, but the chloroform was discontinued after four inhalations. At the time of the visit, a distinctly perceptible tremor, rather than a well characterised muscular contraction, pervaded the entire body, interrupted at intervals of an hour or two, by violent though transient spasms. Muscles of the jaws but slightly affected; he converses without much difficulty though the effort is apt to excite convulsive twitches of the muscles concerned in deglutition. Continue the prescription of yesterday, and let him take in addition

℞. Sulph. quiniæ, gr.ii, every two hours.

Brandy and arrow-root as nourishment, p. v. n.

22d.—Patient remained in the same state as at last visit, during the greater part of yesterday. At 8 P.M., after awaking from a restless and disturbed sleep, tetanic contractions supervened of a more violent and aggravated form, but soon remitted into a slight vibratory motion of the muscles.

Present condition.—Trismus more marked than at last visit, yet he is still able to swallow liquids without much inconvenience. Pulse 108, soft and compressible; respiration natural; skin moist and warm. Bowels have not been moved since the exhibition of the Ol. Ricini, at the onset of the disease.

Ordered—℞. Olei croton Tiglii, gtt. ij,

Olei Ricini, ℥j.

M.

To be repeated, if necessary.

Also, an injection of fol. tabaci, in the proportion of two ounces of the tobacco to one pint of boiling water.—Cont. alia.

The tetanic symptoms gradually increased in severity till between 2 and 3 o'clock, P.M., when he expired. His mind continued clear, and his mental faculties unimpaired in vigor, until within about half an hour of his death.

*Sectio-Cadaveris twenty-one hours after death.*—Whole body, especially the abdomen, much swollen and very tense. Muscles of neck and abdomen rigidly contracted, while those of the arms and legs were in a normal state. The wound of the hand partially cicatrized, but with a superficial slough adhering to its surface, and emitting a strong and offensive odor. Tracing the course of the nervous irritation upwards to the cerebro-spinal axis, the nervous trunks were found remarkably vascular; minute vessels could be seen ramifying, in a beautifully arborescent manner, over the entire circumference, imparting an uniform pink tinge to the exterior. Upon making a section of the nerve, its structure was found, to all appearance, perfectly healthy, both as regards color and consistence. The nerve endowing the little finger, was completely but irregularly divided by ulceration at the site of the wound; at the cardiac extremity of the division, existed a bulbous enlargement, three-fourths of an inch in extent, elliptical in shape, and compressed laterally; the flattening seemed the result of pressure from a fragment of rock, the size of a small pea, resting directly upon the diseased nerve. The exterior of the tumor was of a grayish pink color, mottled with black, from the grains of powder deeply imbedded in the surrounding textures; the interior resembled the ordinary nervous tissue, each filament slightly separated from the adjoining, and the interstices filled with a yellowish substance, probably adipose.

*Spinal Column.*—The spinous processes and a part of the laminæ of the vertebræ having been removed, a pretty uniform layer of coagulated blood, one and a half lines in thickness, was discovered extending from opposite the axis to about six inches below the vertebra prominens. At the other parts of the extent of the spinal canal, the coagula no longer existed, but the surface presented an extremely congested appearance. Upon making a longitudinal section of the dura mater, the spinal cord presented a beautiful net-work of enlarged and tortuous vessels coursing their way over its exterior; the net-work was most apparent opposite the origin of the brachial plexus and at the commencement of the cauda equina. Internally the cord was of a healthy appearance.

*Encephalon.*—Upper part and base of the brain slightly congested; no effusion of serum or pus; sinuses distended with blood. At the base of the brain, blood to the amount of perhaps one drachm was effused, occupying chiefly the posterior subarachnoidean space.

I may state that the propriety of amputation of the arm was discussed, at the time Prof. Eve was first made aware of the existence of tetanus, but inasmuch as the disease had already existed twenty-four hours, and had involved the entire arm and forearm, resort to the knife was deemed inexpedient. As all the details, even the most trivial, appertaining to a disease with whose pathology we are so slightly acquainted, must be of importance, I will conclude this sketch, already perhaps too much extended, by an account of the weather during the progress of the disease. On the day upon which the accession of the tetanus occurred, the wind was south west; in the forenoon some rain fell, but soon ceased, though the sky continued overcast and the atmosphere warm and damp. Upon the following day the sun shone out brightly, giving fair but deceptive promise of fine weather; for until the fatal termination of the case, it continued most variable, though in the main, clear and pleasant. The course of the wind was not particularly noted, though it was generally observed to be from the north west.

January 25, 1851.

